# Cost analysis of implementing a community health worker-led weight reduction randomized-controlled trial among prediabetic south asian patients at primary care sites in NYC

**DOI:** 10.1186/s13012-025-01439-2

**Published:** 2025-06-02

**Authors:** Avni Gupta, Laura C. Wyatt, Shinu Mammen, Jennifer M. Zanowiak, Sahnah Lim, Nadia S. Islam, Rashi Kumar, Susan Beane, Heather T. Gold

**Affiliations:** 1 Department of Public Health Policy and Management, New York University School of Global Public Health, New York, NY USA; 2 Present Affiliation: Researcher, Health Care Coverage and Access, The Commonwealth Fund, New York, NY USA; 3 Department of Population Health, NYU Grossman School of Medicine, New York, USA

**Keywords:** Cost analysis, Diabetes prevention, Implementation costs

## Abstract

**Background:**

We conducted a cost analysis of implementing a randomized controlled trial that proved the effectiveness of a community health worker (CHW) facilitated weight loss intervention among South Asian patients with prediabetes receiving care at primary care practices in New York City. South Asians have a high prevalence of diabetes, but no study to date has evaluated the cost of implementing an evidence-based lifestyle intervention in this population. Cost estimates are necessary for an intervention’s adoption and scale-up.

**Methods:**

The first wave of the intervention was implemented in-person, followed by two waves implemented remotely during the COVID-19 pandemic. We estimated the implementation, intervention, and adaptation costs and the costs by each wave of implementation, by applying the Gold et al.’s economic framework and ERIC discrete implementation strategy compilation Costs were calculated from the perspective of a health care payer, public health agency, or health care system. The CHW intervention included group education sessions over six months. For each wave, we separately estimated the total cost, cost per practice, and cost when implemented at only one practice. Using the Bureau of Labor Statistics salary estimates, we calculated the national average (mean salary) and lower (25th percentile salary) and upper (75th percentile salary) bounds.

**Results:**

The average total 6-month implementation costs over 3 waves, each targeting seven practices was $215,420 (range: $158,620-$257,020). Program staff salaries comprised > 93% of total costs. Adaptation cost was nearly 1/3 of start-up costs. On average, implementation at one practice would cost twice as much as the per-practice costs when implemented simultaneously at seven practices in a wave, due to spread of start-up costs across multiple sites.

**Conclusions:**

Staff salaries comprise most of the budget to implement such an intervention. It is most efficient for an agency to implement this intervention across several practices simultaneously. Decision-makers will need to evaluate relative costs and effectiveness of other options to achieve weight loss in a minority community with constrained resources.

**ClinicalTrials.gov:**

This study was registered on June 15, 2017 at https://www.clinicaltrials.gov as NCT03188094. https://clinicaltrials.gov/ct2/show/NCT03188094.

Contributions to the literature

- Community Health Workers (CHWs) are increasingly implementing evidence-based behavioral interventions, which unlike the traditional clinical interventions, are complicated by the fact that the implementation strategy (CHWs) is an intricate component of the intervention itself (behavior change).
- This paper applied the existing cost and implementation strategies’ frameworks, to measure and report comprehensive cost estimates of implementing a CHW-led, evidence-based behavioral intervention among a high-risk minority community.
- Interventions are also increasingly being implemented remotely however, few studies have reported comprehensive cost estimates to adapt interventions for remote implementation; this paper defines and reports the adaptation costs of a behavioral intervention.

## Background

Overall, one in four health care dollars in the United States is spent on care for people with diagnosed diabetes. The economic cost of diabetes in 2017 was $327 billion, an increase of 26% from 2012 to 2017 [1]. Type II diabetes mellitus (DM) is a leading cause of morbidity and mortality [2]. The Diabetes Prevention Program (DPP), a comprehensive lifestyle management program and a gold standard behavioral weight loss program, has demonstrated clinical and cost effectiveness in achieving weight loss that delays or prevents progression from prediabetes to diabetes [3]. Lifestyle management through physical activity and healthy dietary practices remains a crucial tool for weight loss, even with the advent of newer weight loss drugs which have limited eligibility and insurance coverage, can have adverse effects, and are most effective when taken along with healthy lifestyle management practices [4]. Although DPP has been recommended by several national organizations [5], its adoption in underserved, resourced-limited settings is hampered by the high cost of implementation per participant [3, 6]. Estimates suggest that the cost is around $1399 per year per participant in 2000 US dollars [5, 7]. This high cost can be attributed to the use of specialized health care personnel and individualized approach to deliver the intervention [3]. Several attempts have been made to implement DPP using low-cost approaches such as community-based implementation, use of non-specialized personnel, and group-based delivery [3, 8]. Such translations of DPP implementation have been found effective in achieving weight loss [8]. Zhou et al., in their systematic review of interventions to prevent diabetes, concluded that while both lifestyle and metformin intervention were cost-effective based on an incremental cost-effectiveness ratio (ICER) threshold of $50,000/quality-adjusted life year, lifestyle interventions had lower ICER, and the ICERs of DPP-based lifestyle interventions were nearly half that of lifestyle interventions that did not follow a DPP curriculum [9]. They also found that group-based DPP delivery using a combination of health professionals and lay health workers have lower ICERs than one-to-one delivery or delivery using health professionals [9].

Community Health Workers (CHWs) are increasingly being called on to deliver lifestyle interventions. CHWs are trained members of the public health workforce who are recognized and trusted members of the community and are familiar with the local resources available for community members [10–12]. They serve to bridge the cultural and literacy gaps between communities and health care systems, by providing culturally congruent and personalized peer-coaching [10–12]. CHW interventions have demonstrated promising results in improving health knowledge, behaviors and outcomes, in particular for underserved communities [10, 13].

Given CHWs’ ability to bridge these gaps, and because diabetes disproportionately affects South Asian Americans [14], it has become increasingly important to scale-up the implementation of programs that can prevent DM in this population subgroup. Both nationally and in New York City (NYC), rates of prediabetes [2] and DM itself are higher among South Asian Americans compared to non-Hispanic White adults [14, 15]. Further, South Asian Americans have an increased risk for developing DM at a lower body mass index compared to other racial and ethnic groups [14]. Lifestyle interventions may be especially important to address complex behavioral processes under resource-limited conditions among this group, with their limited English proficiency and lack of ability to access culturally appropriate community resources [14]. Several DPP interventions, including those delivered by CHWs conducted among South Asian immigrants in high-income countries, have shown positive dietary changes and increased physical activity, leading to a modest weight loss and reduction in the incidence of DM [14].

The Diabetes Research, Education, and Action for Minorities (DREAM) Initiative is a theory-driven 5-year randomized controlled trial intervention adapted from the DPP that uses group-based delivery and non-specialized personnel, CHWs, as an implementation strategy for delivering DPP lifestyle interventions to support weight loss among at-risk South Asian American patients receiving care at primary care practices in NYC. The primary outcome of the DREAM intervention was a ≥ 5% weight loss at 6-month follow-up as compared to the baseline. Outcome analysis found that when adjusting for the baseline weight, sex and nesting within the primary care site, 18.8% patients in the treatment arm achieved ≥ 5% weight loss vs. 11% in the control arm (*p* < 0.001) [16].

Several studies have demonstrated cost-effectiveness of CHW-led lifestyle interventions to prevent diabetes or in achieving weight loss among different populations, but none included South Asian Americans [17–20]. Moreover, these studies focused on costs associated with clinical utilization and health care expenditures, and did not include the implementation cost. Any adoption decision by policy makers is likely to be driven by the cost of implementation, in addition to effectiveness evidence. Therefore, a lack of comprehensive cost estimates is a barrier to the implementation of evidence-based practices, such as group-based DPP interventions [21].

Two studies have reported program or intervention implementations costs for DPP interventions using CHWs as the implementation strategy. Yeary et al. conducted a cost analysis of a DPP intervention adapted for Black communities implemented in churches in Arkansas [5]. Lawlor et al. reported the direct medical costs, which included implementation costs, of a DPP intervention delivered by CHWs in North Carolina [3]. To our knowledge, there are no economic evaluations of CHW-driven DPP interventions among South Asian Americans. Moreover, these studies were implemented in-person and hence have limited applicability in the post-pandemic setting in which remote implementation has gained acceptance and prevalence. Remote implementation of lifestyle interventions was increasing even before the pandemic, as noted in a review of cost effectiveness of diabetes prevention interventions [9]. Also, both of those interventions were based in rural areas, unlike DREAM, which was implemented in an urban area.

The goals of this economic evaluation were to estimate the start-up cost of a CHW-driven group-based lifestyle intervention for South Asian Americans, the cost of adapting the in-person intervention for remote implementation, and to compare the cost of implementing at different scales. Additionally, we estimate the per-capita cost of implementing our CHW-led group-based community-level DPP intervention and calculate cost-effectiveness estimates.

Stakeholders, such as health care payers, public health departments, Accountable Care Organizations, health care systems, and practice-based research networks, that are interested in preventing diabetes in high-risk minority populations residing in an urban area, would benefit from results from this cost analysis. More broadly, our cost analysis will be of value to the stakeholders who wish to facilitate weight loss in urban dwelling, underserved, high-risk racial and ethnic minority communities. Our cost analysis will contribute to the larger literature on economic evaluations in implementation research, whose importance is widely acknowledged by implementation frameworks [22]. While such evaluations have improved over time, these analyses are scarce, with existing studies typically lacking sufficient detail about the costs associated with implementing new interventions and relying on data collected retrospectively after implementation has occurred [23]. We hope to provide an example of collecting and analyzing data for conducting economic evaluations on program implementation costs, as the existing frameworks typically offer little direction for such cost analysis [23]. Findings from our study can help inform value propositions of some of the recent initiatives and programs that the Centers for Medicare and Medicaid Services’ Innovation Center has proposed, such as the States Advancing All-Payer Health Equity Approaches and Development (AHEAD) Model and the Making Care Primary (MCP) Model. Aimed at advancing health equity, these models emphasize the need to integrate access to community resources for beneficiaries [24].

## Methods

### Study setting and design

The DREAM Initiative is a partnership among New York University Grossman School of Medicine; Healthfirst, a not-for-profit Health Maintenance Organization serving over 35,000 South Asians in NYC; Healthify (now WellSky), a leading software provider to health plans, hospitals [25], and provider networks that work in low-income communities; and South Asian community organizations including the DREAM coalition, India Home, United Sikhs, Council of People Organization, and the South Asian Council on Social Services. Although the detailed design and procedures for DREAM have been reported elsewhere [14], we provide a brief background of the study and then describe the processes for the cost analysis. The study is a two-arm Randomized Controlled Trial that leverages CHWs as an implementation strategy to test the implementation and effectiveness of a multi-component intervention to facilitate weight loss among South Asian Americans at risk for diabetes.

Seventeen primary care practice sites were selected throughout the NYC area that were in Healthfirst’s network. On average, nearly 80% of patients seeking care at these clinics were South Asian Americans and only about 16% used English as their primary language. These clinics largely served Medicaid patients (on average, 77% of patient care revenue came from Medicaid reimbursements).

At each primary care practice site, eligible patients were randomly assigned to the treatment or control group. The treatment group was contacted, screened and consented by CHWs to receive the DREAM intervention. The DREAM protocol consisted of five monthly, 60-min, CHW-led group education sessions over a 6-month period; session topics included diabetes management and prevention, diet/nutrition, and physical activity to promote weight loss and prevent DM. This standardized curriculum on DM prevention from the DPP was culturally and linguistically adapted for South Asian Americans. The sessions were based on the principles of adult learning techniques and group-based learning.

Seventeen primary care practice sites were divided into three groups, and the intervention was implemented in three successive waves by group – seven sites in wave 1, seven sites in wave 2, and six sites in wave 3 (two CHWs were at one site, stratified by gender of participant). The intervention was designed such that one CHW was assigned to deliver the intervention to all patients at one practice site. The patient load of each CHW was similar, ranging from 17—32 (average 24) in wave 1, 16–28 (average 21) in wave 2, and 14–26 (average 24) in wave 3. Although the intervention was designed to be implemented in-person, after the first wave (January 2019 – July 2019), the intervention implementation was switched to fully remote to meet social distancing requirements enforced during the COVID-19 pandemic. Therefore, before implementing wave 2 (January 2020 – July 2020) and wave 3 (March 2021 – February 2022), the intervention was adapted for remote implementation. CHWs delivered education sessions in primary care practice offices or other community paces in wave 1, and through phone or virtual video platform in waves 2 and 3. Between sessions, CHWs followed-up with participants bi-weekly to engage them in goal setting related to weight loss and healthy lifestyle changes and to review progress on established goals. The primary end point of the study was achievement of ≥ 5% weight loss in each patient, and we found (unpublished) that an additional 8.7% of patients achieved this outcome in the treatment arm compared with the control arm. The study has been approved by the New York University Langone Institutional Review Board.

### Data sources

We used the economic framework for implementation studies proposed by Gold et al., to guide our cost measurement and reporting [21]. Based on this framework, we categorized all costs into implementation costs, intervention costs, and adaptation costs. We further applied the ERIC discrete implementation strategy compilation to identify the implementation costs [26]. The ERIC’s list includes a comprehensive list of possible implementation strategies based on an expert panel review. We mapped the activities and tasks involved in implementing our study to these strategies to determine which activities and tasks should be considered implementation costs. Below we describe the different types of costs considered for this analysis.

#### Implementation costs

All the start-up costs were included in this category and were associated with training, equipment, and recruitment, in alignment with ERIC’s list elements [26], such as, “Build a coalition” by recruiting and cultivating relationships, “Change physical structure and equipment”, “conduct educational meetings” to teach stakeholders about the intervention, and “Conduct ongoing training.” The program coordinator and the CHW manager tracked and calculated all start-up costs in a spreadsheet. It included all equipment purchased for CHWs, primary care practice site recruitment, and Electronic Health Record training (program staff time for recruiting primary care practice sites and for conducting trainings for primary care clinicians), patient recruitment (program staff time for pulling the list of patients from the Electronic Health Record, and mailing recruitment letters to intervention patients) and CHW training (program staff time in conducting trainings for CHWs on the contents of the curriculum and implementation of the intervention, and CHWs time in attending the trainings). Total start-up costs excluded time costs for recruiting CHWs, because the DREAM initiative was implemented in a community where the research staff had established relationships with existing CHWs from prior projects. Time to develop the curriculum and translate it into different languages spoken by the target community were not included in implementation cost analysis, because future intervention implementations will not incur these costs.

#### Implementation and Intervention costs in Waves 1, 2 and 3

Across all waves there were implementation and intervention costs. CHWs used REDCap [27, 28] and program staff used spreadsheets to document time spent on different activities. CHWs’ time to conduct screening calls, and obtaining consent (ERIC’s list elements such as, “Assess for readiness” and “Prepare patients/consumers to be active participants”) [26], and program staff’s time spent in all supervision, support, and coordination of meetings among staff, including with CHWs, in performing administrative work, and in reviewing and editing the curriculum before each session to meet specific just-in-time needs were also included in intervention costs were considered implementation costs (ERIC’s list elements such as, “Provide clinical supervisions”, “Provide Local technical assistance”, “Organize clinician implementation team meetings” “Provide ongoing consultation”, “Tailor strategies”, and “Promote adaptability”) [26]. Intervention costs included CHWs’ time spent in completing the intake survey for baseline goal setting, planning and delivering education sessions, the bi-weekly meetings/calls with participants and unscheduled encounters where patients contacted the CHW for additional support. The time estimates were recorded either immediately upon completion of the meeting or retrospectively by CHWs or program staff. Research costs, such as costs for ethics review board approvals, research meetings, or research-specific documentations, were not included, because they would not be part of a standard program implementation. In waves 2 and 3, no time was attributed to CHWs preparing for in-person education sessions such as for arranging chairs, setting up screens, or cleaning the space, because sessions were virtual. In addition, in waves 2 and 3, weekly virtual group meetings among CHWs moderated by the CHW Manager were introduced to complement in-person one-on-one weekly meetings of CHWs with the Program Coordinator.

#### Adaptation costs

Before the intervention could be switched to fully remote, adaptation was required. Based on estimates from the Program Coordinator, CHW Manager, and CHWs, we documented time spent in technology orientation (developing remote video platform guides, CHW self-training, and patient training), in adapting curriculum for virtual delivery (reviewing, streamlining and shortening presentations, and developing fidelity checklists), in developing supplemental videos to supplement education sessions (review of dietary guidelines, resources to develop scripts, video recording, and editing), in developing and translating community resource guides for patients to achieve lifestyle goals amid the pandemic, and for buying and mailing equipment (weight scale and curriculum) to each patient.

### Analytic approach

Defining a cost analysis perspective is important to estimate relevant resources across implementation phases [29]. We calculated costs from the perspective of a third-party payer (such as a health insurance company, a public health agency, or a healthcare system) that values or has a financial incentive to reduce DM incidence or achieve weight loss in a minority underserved population.

Implementation occurred from 2019–2022, and costs use 2019 wage estimates. Data for staff salaries were obtained from the 2019 data of the US Bureau of Labor Statistics (BLS) [30]. We estimated salary costs for three types of program staff who were key to protocol implementation: Program Coordinator (BLS occupation: Health Education Specialist), CHW Manager (BLS occupation: Community Health Worker, Social and Community Service Manager) and CHW (BLS occupation: Community Health Worker). Using the national wage and a 30% fringe rate assigned to the job classification, a dollar amount was assigned to each hour of staff time. We used the national average wage as an estimate of average costs, the 25th percentile of wage nationally as the lower bound, and the 75th percentile of wage nationally as the upper bound (Table 1).

**Table 1:** Compensation rates for occupational classifications required for study implementation—Bureau of Labor Statistics 2019

	Program coordinator wage + 30% fringe rate	CHW manager wage + 30% fringe rate	Community Health Worker wage + 30% fringe rate
Mean	$35.05 + $10.52 = $45.57	$29.09 + $8.73 = $37.82	$21.34 + $6.40 = $27.74
25th percentile	$25.23 + $7.57 = $32.80	$19.70 + $5.91 = $25.61	$15.79 + $4.74 = $20.53
75th percentile	$42.39 + $12.72 = $55.11	$36.08 + $10.82 = $46.90	$25.41 + $7.62 = $33.03

We estimated the total costs to operate the program over a 6-month period for each wave. Even though in wave 3 only 6 primary care practice sites participated (vs. 7 sites in wave 1 and wave 2), for comparability, we used a multiplier of 7 for wave 2 cost calculations. We also estimated the cost per practice and the cost assuming the intervention was implemented at only one practice, considering that the costs associated with the trainer’s time for training CHWs will not change with the scale of the implementation. The per-practice estimates can guide program financers about the number of practices that can be targeted given their budget and an individual CHW’s capacity. All estimates are based on a setting where one CHW works with all patients at one practice with a caseload of 24 patients for a 6-month implementation period, and one trainer was required to train all CHWs. Costs were calculated separately for in-person implementation using data from wave 1 and for remote implementation using data from waves 2 and 3. All costs were rounded to the nearest $100 or to nearest $10 if the costs were less than $100. We then calculated the cost of implementing per-capita (or per-patient) by dividing the total cost by the total number of patients across three waves and seven practices (24 patients X 7 practices X 3 waves = 504 patients). Finally, we calculated the incremental cost-effectiveness ratio by dividing the difference in costs between treatment and control arms (cost for the control arm was assumed zero) by the difference in effectiveness (percentage achieving target weight loss) between treatment and control arms, yielding the additional cost required to achieve 1 percentage point increase in patients achieving target weight loss.

We used the STROBE checklist for cross sectional studies as a reporting guide (Appendix).

## Results

Table 2 shows the estimated costs using national average, 25th percentile and 75th percentile compensation rates. The total cost was nearly $186,020 based on national average compensation. This included nearly $80,300 of implementation costs, $88,420 of intervention costs and $17,300 of adaptation costs. The total cost could range from nearly $137,420 in regions offering salaries at 25th percentile to nearly $221,420 in regions with compensation at 75th percentile. Intervention and implementation costs were estimated $40,000 for wave 1 vs. $49,320 for wave 2 vs. $51,400 for wave 3 using national average rates. In addition, 93.8% (ranging from 92.6% to 94.4%) of the total costs incurred were attributable to personnel compensation. Start-up costs, a part of implementation costs, are lower than the cost of any one wave, either in-person or virtual; using national average wages, the start-up costs were $28,000. In each wave, the cost of the actual intervention was higher than the cost to implement it. Table 3 compares the costs of implementing per practice and if implemented at only one practice. Data suggest economies of scale such that if one practice implements this intervention, the costs will be 40% higher than the per-practice costs incurred when implemented across seven practices. The per-capita cost of the intervention was $369 when implemented across seven practices. It cost nearly $21,381 ($15,819 to $25,450 using 25th and 75th percentile salary estimates, respectively) to achieve an additional 1%-point increase in patients meeting the target of ≥ 5% weight loss.

**Table 2:** Total costs of implementing a CHW-led weight loss intervention across seven primary care practices using national average, 25th percentile and 75th percentile salary estimates in 2019 US$

Cost category	Type of cost	Average	25th percentile	75th percentile
Start-up costs				
CHW training – trainees	Implementation	10,200	7,500	12,100
CHW training – trainers	Implementation	8,100	5,700	10,000
Pc recruitment, training	Implementation	1,400	1,000	1,700
Patient recruitment	Implementation	3,000	2,200	3,700
Equipment for CHWs	Implementation	5,300	5,300	5,300
Wave 1 (In-person)				
Screening	Implementation	300	200	300
Intake survey	Intervention	3,500	2,600	4,200
Education sessions	Intervention	10,200	7,600	12,200
Bi-weekly follow-ups	Intervention	12,000	8,600	13,800
Unscheduled encounters	Intervention	300	200	400
Supervision	Implementation	10,600	7,600	12,900
Administrative	Implementation	2,100	1,500	2,600
Curriculum adaptations	Implementation	1,000	700	1,200
Wave 2 (Remote)				
Screening	Implementation	300	200	400
Intake survey	Intervention	2,900	2,100	3,400
Education sessions	Intervention	16,300	12,100	19,500
Bi-weekly follow-ups	Intervention	11,000	8,000	12,900
Unscheduled encounters	Intervention	20	20	20
Supervision	Implementation	15,700	11,400	18,900
Administrative	Implementation	2,100	1,500	2,600
Curriculum adaptations	Implementation	1,000	700	1,200
Wave 3 (Remote)				
Screening	Implementation	400	300	500
Intake survey	Intervention	2,900	2,100	3,500
Education sessions	Intervention	15,900	11,800	19,000
Bi-weekly follow-ups	Intervention	13,400	9,900	16,000
Unscheduled encounters	Intervention	0	0	0
Supervision	Implementation	15,700	11,400	18,900
Administrative	Implementation	2,100	1,400	2,600
Curriculum content edits	Implementation	1,000	700	1,200
Adaptation cost				
Technology orientation	Adaptation	5,300	4,000	6,300
Curriculum adaptations	Adaptation	300	200	400
Supplemental videos	Adaptation	2,000	1,400	2,300
Resource guides	Adaptation	3,600	2,600	4,400
Equipment	Adaptation	6,100	4,900	7,000
Total start-up cost		28,000	21,700	32,800
Total implementation cost (wave 1)		40,000	29,000	47,600
Total implementation cost (wave 2)		49,320	36,020	58,920
Total implementation cost (wave 3)		51,400	37,600	61,700
Total adaptation cost		17,300	13,100	20,400
Total personnel cost		174,620	127,220	209,120
Total cost		186,020	137,420	221,420
Personnel cost as percent of total cost (%)		93.8	92.6	94.4
Using Gold et al.’s Framework				
Total Implementation costs		80,300	59,300	96,100
Total Intervention costs		88,420	65,020	104,920
Total Adaptation costs		17,300	13,100	20,400

Ranges based on occupation type from the 2019 U.S. Bureau of Labor Statistics. Rounded to the nearest $100 s or to nearest 10 s if number was < 100

**Table 3:** Comparison of total, per practice and one practice costs using national average, 25th percentile and 75th percentile salary estimates in 2019 US$

	Mean			25th percentile			75th percentile		
	Total cost	Per practice	1 practice	Total cost	Per practice	1 practice	Total cost	Per practice	1 practice
Total start-up cost	$28,000	$4,100	$7,300	$21,700	$3,100	$5,400	$32,800	$4,600	$8,600
Total implementation cost (wave 1)	$40,000	$5,580	$6,480	$29,000	$4,160	$4,760	$47,600	$6,990	$7,990
Total implementation cost (wave 2)	$49,320	$6,850	$7,750	$36,020	$5,030	$5,630	$58,920	$8,450	$9,450
Total implementation cost (wave 3)	$51,400	$7,300	$8,200	$37,600	$5,350	$5,950	$61,700	$8,880	$9,880
Total adaptation cost	$17,300	$2,550	$7,900	$13,100	$1,930	$5,800	$20,400	$2,860	$9,400
Total cost	$186,020	$26,380	$37,630	$137,420	$19,570	$27,540	$221,420	$31,780	$45,320
Ratio of 1 practice to per practice cost	1.4			1.4			1.4		

## Discussion

Our findings showed that a large-scale implementation of DPP intervention culturally and linguistically adapted to South Asians was more efficient than a small-scale implementation due to economies of scale and a single CHW being able to work across several sites once trained and prepared; this implementation approach reduced the cost of implementation per practice in urban primary care clinics to about half of what it would cost to implement at one practice. Cost-per-practice for a 6-month long implementation across seven practices was $26,380 vs. an estimated $37,630 for one practice (2019US$). More than 90% of this cost was attributed to salary expenses. The per-practice metric suggests that it might be unaffordable for a single clinic to implement such an intervention for its patients. However, a third-party payer, healthcare system, primary care practices, provider practice networks, Accountable Care Organizations, or public health agency could achieve greater efficiency by implementing at several practices. Based on a separate outcome analysis [16] that found 8.7% more patients achieved the target weight loss in the treatment group compared with the control group, we estimated it will cost nearly $25,000 to get an additional 1% of the patient population to achieve target weight loss; additional work could inform the population benefits beyond achieving ≥ 5% weight loss and the ultimate decision of whether to implement this intervention. It should be noted that the percentage of patients achieving the primary goal of ≥ 5% weight loss at 12 months from baseline were higher in the treatment arm but not significantly different from control arm (13.9% vs. 8.5%; *p* = 0.089). Although not included, the program also incurred some development costs of nearly $30,000 at national average compensation rates for developing and translating the curriculum, educational handouts, participant baseline survey or the intake form, participant follow-up surveys, and recruitment letters and flyers. These costs were not included in our implementation cost analysis as future implementation of the intervention will likely not incur these costs.

We found that intervention implementation will cost nearly $369 per-patient when implemented across seven practices. This estimate is comparable to Yealy et al.’s cost analysis of CHW-driven DPP intervention in a Black community [5]. They found their total cost per-participant to be $348.95 in 2014; after updating for inflation, their cost was $383.35 in 2019. Both our findings and Yearly et al.’s findings show that leveraging CHWs as an implementation strategy to deliver lifestyle components of DPP might cost less than implementing these interventions individually through other types of professionals [6]. Another cost analysis of DPP by CHWs in 2010 found that the total per capita direct medical cost over the two years of the trial in the lifestyle group was $850; after updating for inflation, their cost was $987.39 in 2019 [3]. We did not capture medical costs in our analysis. A systematic review found that when lay healthcare professionals are included in the implementation, the cost of implementing diabetes prevention interventions is less than half than when delivered through healthcare professionals [9]. This review also noted that DPP-based interventions incur lower costs than non-DPP based approaches [9]. In addition, they found that high-risk population approaches are more efficient than population-based approaches by having a stronger effect [9]. DREAM targeted a high-risk population and tested a group-based DPP intervention implemented by lay healthcare professionals. Our ICER estimate is nearly $25,000 for an additional 1 percentage point increase for patients to achieve ≥ 5% weight loss. A standard threshold indicating an intervention is high value is an ICER of $50,000 per Quality-adjusted life year [31]. Although we did not calculate Quality-adjusted life years, based on this threshold, DREAM intervention might be cost-effective when implemented across at least seven practices. Overall, our findings support the general literature suggesting efficiency of group-based non-health professional driven delivery of DPP.

We found that remote implementation incurred higher costs than the in-person wave. Zhou et al., in their review of cost-effectiveness of diabetes prevention interventions found that in recent years, the adoption of virtual media interventions has increased because it enhances access for hard-to-reach populations with mobility issues [9]. They concluded that few studies have evaluated the cost effectiveness of virtually delivered interventions, and the limited evidence shows that they are less cost-effective than in-person lifestyle programs [9]. They suggest rigorous studies to assess cost effectiveness of virtually implemented programs. It should be noted that although in our cost analysis we found remote implementation to cost more than in-person implementation, the context in which we switched to remote implementation was that of a pandemic when the social and emotional needs of the community members were unprecedentedly high. This is supported by our finding that the key cost categories in remote waves that were higher than the in-person wave were ‘education sessions’ and ‘supervision’, suggesting more time spent in 1:1 sessions between CHWs and patients (where social and emotional needs were often the focus of discussion), and between CHWs and their supervisors (where CHWs brought clients concerns to supervisors and collaboratively developed strategies and tools to address these needs; and then CHWs connected patients with community resources to address their needs). Thus, the cost data from our study cannot be used to support or refute Zhou et al.’s findings about lower cost-effectiveness of virtual implementation. We also found that the second remotely implemented wave cost more than the first remote wave. Typically, we would expect a learning curve and reduced costs over time. Between the two remote waves, the cost category of ‘biweekly follow-ups’ was higher in the later wave. CHWs reported spending longer times per patient in wave 3 than wave 2. This was likely driven by discussions about COVID-19 vaccination during Wave 3, which increased the duration of follow-up calls. In addition, the CHWs spent more time for eligibility screening calls during Wave 3 than during Wave 2. This could be a result of the recruitment timing for the two waves. Wave 2 recruitment was conducted before the pandemic, but Wave 3 recruitment was conducted during the peak of the pandemic. We encourage future studies to conduct such comparisons and help build a greater understanding of remote implementation strategies.

Although adaptations of evidence-based practices for optimizing their implementation are widespread, little attention has been given to the economics of adaptation [32]. Adaptation has resource implications and hence economic implications [32]. Our study fills an important gap in the economic evaluation of adaptations by reporting the cost of an unplanned or reactive adaptation of the intervention that was originally designed to be in-person into a fully remote implementation. In response to the lock-down orders during the COVID-19 pandemic, we adapted the intervention but not the implementation strategy. This necessitated training CHWs to deliver the intervention remotely, in addition to adapting the intervention materials for remote use.

Several studies have demonstrated that CHW-led interventions among patients with chronic diseases achieve cost savings for healthcare payers such as Medicaid based on medical costs. Our study did not focus on medical costs. However, very few studies provide a comprehensive analysis of program implementation costs which is necessary to “make wise decisions in the allocation of scarce resources” [33]. Our implementation cost analysis addresses this knowledge gap. Our study makes a unique contribution by analyzing each type of cost, and by providing discrete analysis of costs associated with intervention, implementation, and adaptation.

With the advent of the patient-centered medical home model, primary care practices work increasingly to coordinate care for patients, and there is growing evidence that CHWs can help improve care for patients at these practices. With the Affordable Care Act’s explicit promotion of the inclusion of CHWs as members of the health care team, such implementation cost analysis will become critical inputs to the decision tree models of payers. Current payment and service delivery models are increasingly focused on value-based care, which aim to compensate the providers for delivering care with value for patients, replacing traditional fee-for-service model. Some of the recent models implemented by the CMS Innovation Center, such as Making Care Primary (MCP) Model and the Advancing All-Payer Health Equity Approaches and Development (AHEAD) Model focus on strengthening primary care through integration and care coordination, and emphasize the importance of screening and referral to community-based services and supports, particularly for underserved beneficiaries [24]. As CHWs become incorporated into such team-based models, our study illustrates one approach to implement such integration. To support these models and make a business case, reimbursement structure and policies will be important. The New York State implemented their Medicaid CHW reimbursement policy in 2023. According to this, reimbursement ranges from $35.00 to $12.25 depending on the size of the group (with $35 reimbursement provided for a 30 min individual session) [34]. Maximum allowance per year is 12 units per member, which means that the maximum reimbursement can be $420 per member. In our intervention, the cost of delivering five 1-h CHW-led sessions over 6 months was $369 per patient. Providing such an intervention over 12 months can be covered with the current annual maximum reimbursement of $420 per member per year.

### Limitations and strengths

Several limitations of our analysis must be noted. First, our analysis was limited to quantitative data. An integration of qualitative data can help to better understand the context for implementation of complex interventions [23]. Second, we could not include the cost to recruit CHWs as we had access to an existing pool of trained CHWs from previous studies and the cost to print education materials as printing resources were available through the research institution. Third, our staff cost estimates are based on national average wage rates, which may need to be adjusted to a decision-maker’s local wage rates; we did conduct a broad sensitivity analysis by using the 25th and 75th percentile wages to inform decision-making. Fourth, our cost calculations assumed a patient load of 24 per practice or per CHW. While we have not included the variation in patient load across practices or CHWs for the sake of comparability across waves, it should be noted that the patient load was not fixed across CHWs. Each CHW was working at full capacity with their own patient load, and we averaged the load to 24 patients. This implies that if program implementors seek to target more than 24 patients in a clinic, they might need to hire more CHWs for each practice. It is also important to note that if CHWs are hired to work part-time, the number of CHWs needed to target 24 patients will be higher and hence more training costs might be incurred. Finally, as implemented, we could not disentangle the intervention cost from intervention implementation costs using CHWs as the strategy. It would not have been realistic or ethical to only provide the education materials, i.e., provide the information, without simultaneous CHW facilitation, because previous studies have shown CHW facilitation to be effective.

The following strengths of our analysis must also be noted. We recorded and collected detailed time and cost information concurrently with the program implementation, which makes our estimates less prone to recall bias and useful to decision makers. We report cost estimates separately by comprehensive cost categories including intervention, implementation, and adaptation costs, to facilitate informed evaluation of resources and budget by decision makers interested in implementing this intervention.

## Conclusion

In conclusion, the DREAM CHW-led DPP intervention among seven urban primary care clinics serving South Asian American patients cost approximately double when implemented at a single practice than when scaled up to seven practices. Remote implementation of this intervention was costlier than the in-person implementation, largely due to more time spent by CHWs in education sessions and supervision. The cost to adapt the intervention to remote implementation was about one-third of the start-up cost. As implemented, it will require program implementors to invest nearly $25,000 for 1% of the patients to achieve ≥ 5% weight loss. Program implementors can use this cost data to inform their decisions about implementing weight loss interventions in underserved populations. Future evaluations should compare implementation costs and outcomes across different implementation strategies and interventions, to further facilitate diabetes prevention in underserved populations.

## Data Availability

The datasets used and/or analyzed during the current study are available from the corresponding author on reasonable request.

## Abbreviations

CHW: Community Health Worker
DM: Diabetes mellitus
DPP: Diabetes Prevention Program
ICER: Incremental cost-effectiveness ratio
QALY: Quality-adjusted life year
BMI: Body mass index
NYC: New York City
DREAM: Diabetes Research, Education, and Action for Minorities Initiative
BLS: Bureau of Labor Statistics
